# New-onset atrial fibrillation after percutaneous patent foramen ovale closure: a meta-analysis

**DOI:** 10.1007/s00392-023-02263-8

**Published:** 2023-07-29

**Authors:** Dominik Jurczyk, Sascha Macherey-Meyer, Elias Rawish, Thomas Stiermaier, Ingo Eitel, Christian Frerker, Tobias Schmidt

**Affiliations:** 1 Medical Clinic II, University Heart Center Lübeck, University Hospital Schleswig-Holstein, Ratzeburger Allee 160, 23538 Lübeck, Germany; 2 DZHK (German Centre for Cardiovascular Research), Partner Site Hamburg/Kiel/Lübeck, Lübeck, Germany; 3 Faculty of Medicine and University Hospital Cologne, Clinic III for Internal Medicine, University of Cologne, Cologne, Germany

**Keywords:** Patent foramen ovale, Atrial fibrillation, Percutaneous closure, Occlusion

## Abstract

**Background:**

The exact incidence and predictors of new-onset atrial fibrillation (AF) after percutaneous closure of patent foramen ovale (PFO) are unknown.

**Objective:**

We sought to find post-procedural AF incidence rates and differences due to different screening strategies and devices.

**Methods:**

A systematic search was conducted in Cochrane, MEDLINE and EMBASE. Controlled trials fulfilling the inclusion criteria were included into this meta-analysis. The incidence of new-onset AF was the primary outcome. Further parameters were surveillance strategy, device type, AF treatment and neurological events. New AF was determined as early onset within one month after implantation and late thereafter.

**Results:**

8 controlled trials and 16 cohort studies were eligible for quantitative analysis. 7643 patients received percutaneous PFO closure after cryptogenic stroke or transient ischaemic attack, 117 with other indications, whereas 1792 patients formed the control group. Meta-analysis of controlled trials showed an AF incidence of 5.1% in the interventional and 1.6% in the conservative arm, respectively (OR 3.17, 95% CI 1.46–6.86, *P* = 0.03, *I*^2^ = 55%). 4.7% received high-quality surveillance strategy with Holter-ECG or Loop recorder whereby AF incidence was overall higher compared to the low-quality group with 12-lead ECG only (3.3–15% vs. 0.2–4.3%). Heterogeneous results on time of AF onset were found, limited by different follow-up strategies. CardioSEAL and Starflex seemed to have higher AF incidences in early and late onset with 4.5% and 4.2%, respectively.

**Conclusion:**

Percutaneous PFO closure led to higher AF post-procedural incidence compared to the conservative strategy. Heterogeneity in surveillance and follow-up strategy limited the generalizability.

**Trial Registration:**

Registered on PROSPERO (CRD42022359945).

**Graphical abstract:**

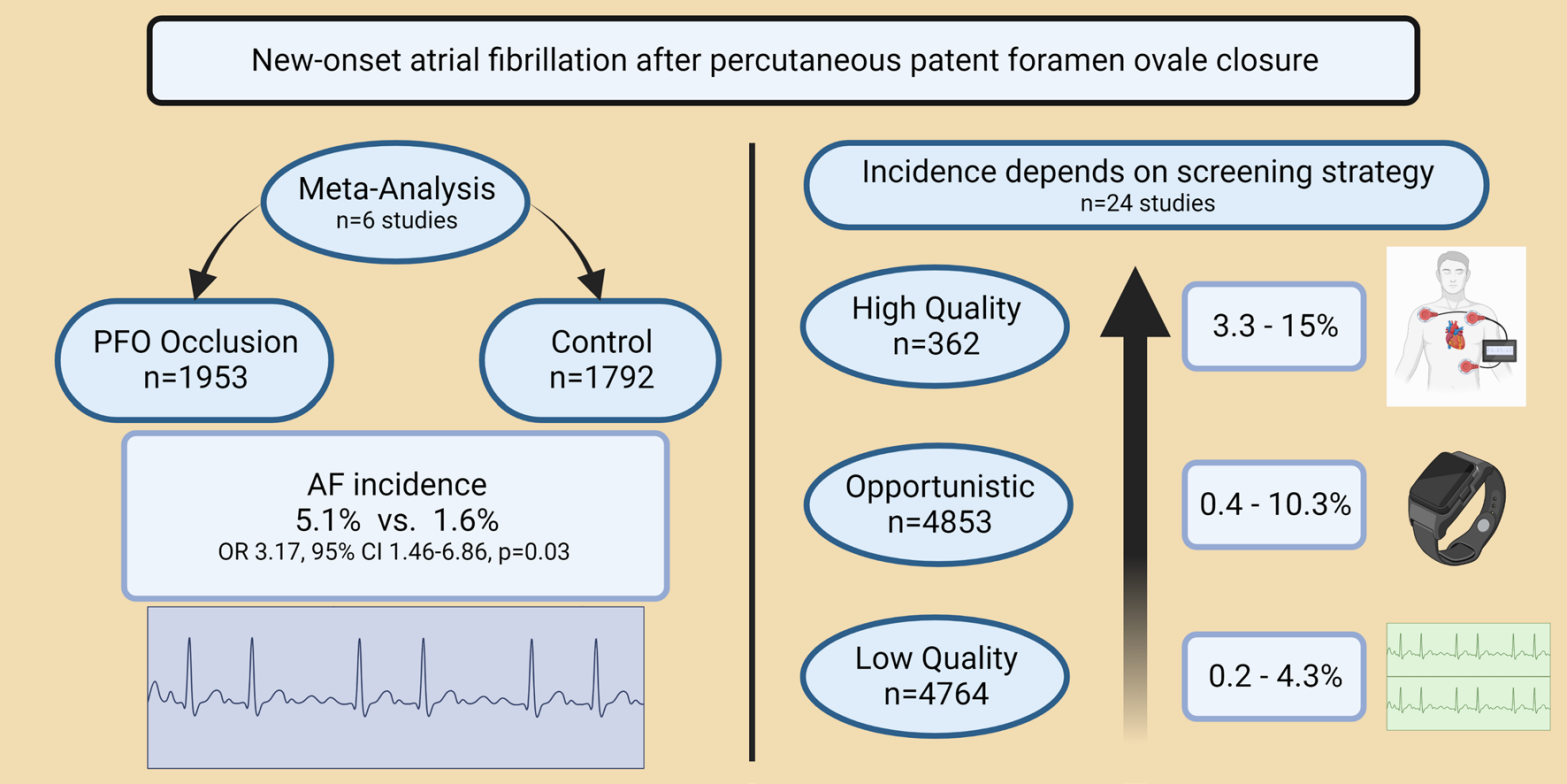

**Supplementary Information:**

The online version contains supplementary material available at 10.1007/s00392-023-02263-8.

## Introduction

The percutaneous patent foramen ovale (PFO) closure has been continuously developed and improved since its first implantation in 1989 by Lock and colleagues [1]. Cryptogenic cerebral ischaemia either defined as stroke or transient ischaemic attack (TIA) constitutes the predominant indication for PFO closure. Diagnosis of cryptogenic ischaemia requires rule-out of atrial fibrillation (AF) by Holter-electrocardiogram (ECG) or continuous monitoring as AF itself is a common cause of cardio-embolic cerebral ischaemia [2]. Given the lower morbidity and cardiovascular risk burden, PFO closure is typically performed in younger patients. Importantly, the first trials reported new onset of AF after percutaneous PFO closure [3, 4]. Several AF predictors, such as male sex, large size and atrial dysfunction, have been described inconsistently in previous studies and failed to demonstrate significance [5–11]. Former meta-analyses focussed on new onset of atrial fibrillation after percutaneous PFO closure regardless of AF screening strategy [12, 13]. In Elgendy et al., an increased rate of AF incidence was found in the early phase of implantation, defined as 45 days after index procedure. In this study, a higher risk seemed to be for the Starflex device, in another for the Gore occluder compared to the Amplatzer [14].

Therefore, our aim was to systematically review studies that reported AF screening strategies and time of AF onset. Treatment and strokes were also examined if data were available.

## Methods

The present meta-analysis was realised according to the PRISMA guidelines, a pre-specified protocol and explicitly reproducible routine for literature search and synthesis [15].

### Search criteria

We performed an electronic search of the bibliographic databases (MEDLINE, EMBASE, and Cochrane Database of Systematic Reviews) and hand-searching of reference lists. We used the following search terms “patent foramen ovale”, “PFO”, “atrial fibrillation” and “AF”, and connected these terms with Boolean operators. The first search was conducted on April 1, 2022. The last search update was carried out on November 21, 2022. No restrictions on publication date or study size were applied.

### Study selection

The study selection was independently performed by two reviewers (DJ, ER). In case of any disagreement, this was resolved by consensus with the senior authors (TS, CF). We included all publications fitting the following inclusion criteria: retrospective, prospective and randomised controlled trials reporting on AF after PFO closure, type of device and AF screening method. Articles published in either German or English were eligible for analysis. Main study reports as well as any supplementary appendices were reviewed.

### Outcome parameters

AF incidence after percutaneous PFO closure was defined as primary outcome of this meta-analysis.

We extracted data on AF incidence from randomised and non-randomised trials and, if available, device-related AF incidence according to AF screening method and subsequent AF management strategies.

### Statistical analyses

Random effects meta-analyses were performed using the Mantel–Haenszel method for dichotomous data to estimate pooled odds ratios (ORs) and confidence intervals (CI). Weights were calculated using Mantel–Haenszel methods. Furthermore, the *I*^2^ statistics to quantify possible heterogeneity was calculated (*I*^2^ < 30%: low heterogeneity; 30% < *I*^2^ < 75%: moderate heterogeneity; *I*^2^ > 75%: considerable heterogeneity; Review Manager 5.4, Nordic Cochrane Centre, Cochrane Collaboration). We defined *P* < 0.05 as a statistically significant difference. The level of evidence of the original trials was evaluated according to the criteria of the Oxford University [16]. To assess the studies’ quality, we judged the individual and overall risk of bias. The risk of bias was assessed by RoB2 tool provided by the Cochrane Collaboration in randomised trials [17]. We used the ROBINS-IAQ8 (Risk of Bias in Non-randomised Studies of Interventions) tool to evaluate non-randomised trials [18]. Two reviewers independently judged the risk of bias according to the given criteria (E. R. and D. J.)

First, we decided to take 45 days as a distinctive cut-off for early or late onset of atrial fibrillation because it was chosen in in the EAPCI position paper [2]. During the study selection and data extraction process, it turned out that most studies chose the first ECG follow-up after one month. Therefore, it was changed for the analysis in the second step to one month. The AF screening method was divided into high quality and low quality. The first included only Holter-ECG and/or external loop recorders (ELR). The latter was at least a structured 12-lead ECG follow-up. Additional symptom-led Holter-ECGs defined an intermediate group of opportunistic AF screening created post hoc.

We did not obtain ethical approval for this meta-analysis because we did not collect data from individual human subjects. The study was registered on PROSPERO (CRD42022359945).

## Results

**Fig. 1:**
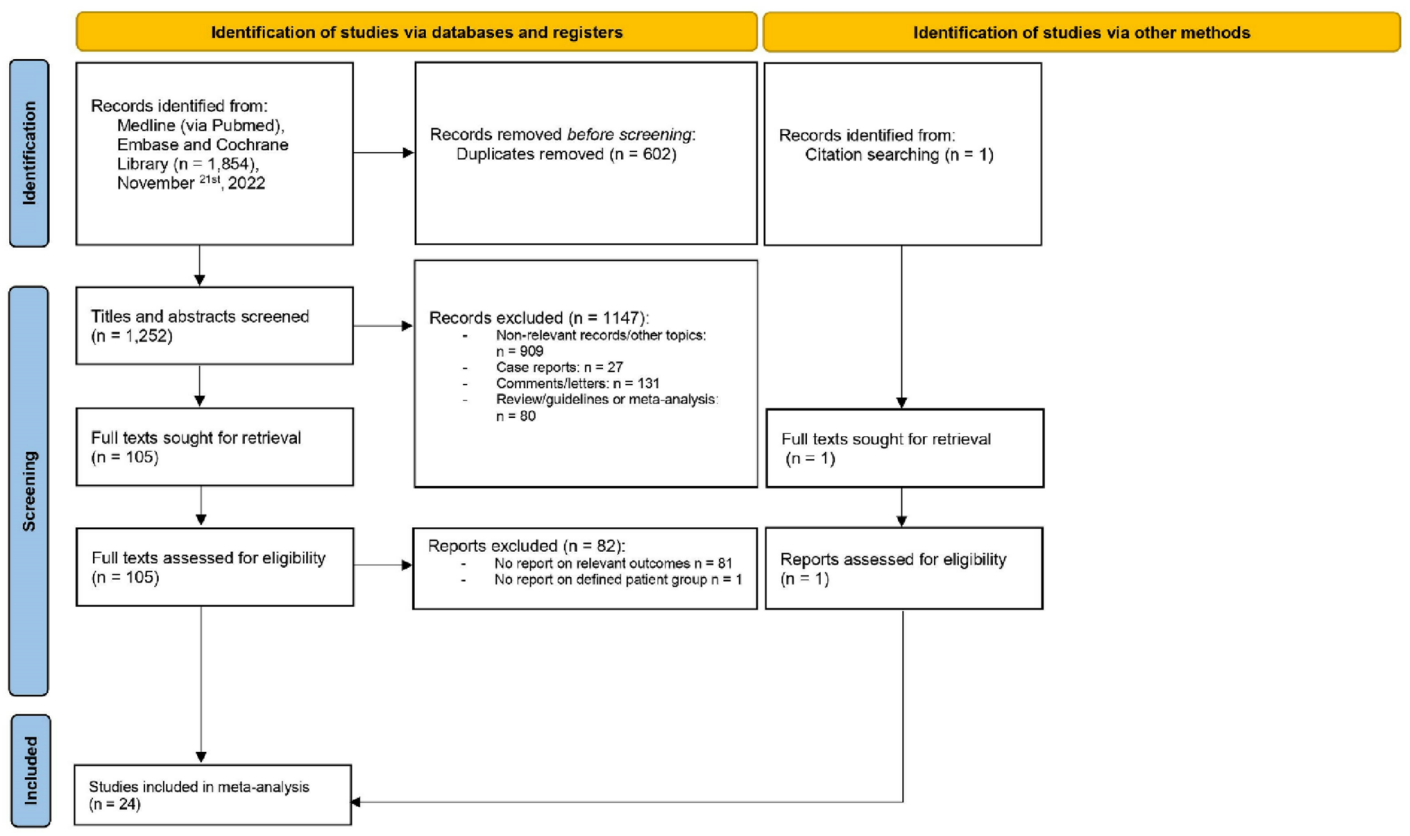
Flow chart. Systematic search in databases and study selection according to the PRISMA guidelines. Reasons for exclusion of studies are given. The last update was on November 21, 2022. Created in Excel, Microsoft Office 365

**Fig. 2:**
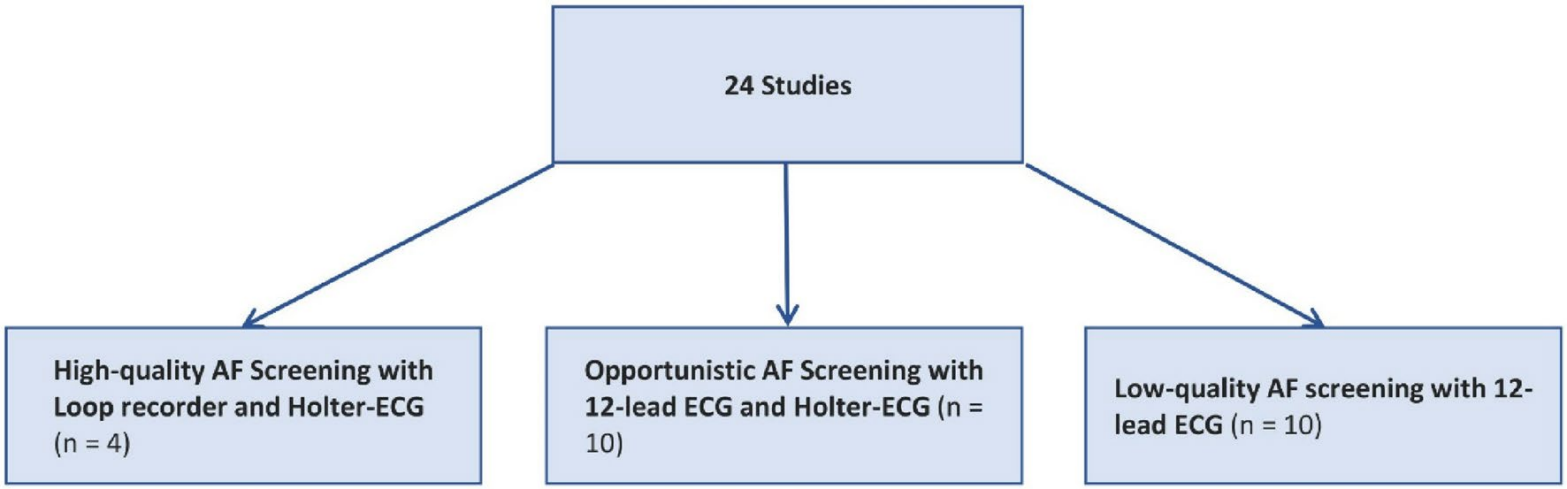
Categorization in AF surveillance groups. All studies were categorised according to quality levels of AF surveillance. The use of Holter-ECG and Loop Recorders is distinctive for high-quality screening. Low-quality screening was sporadic 12-lead ECG only. For studies using a Holter-ECG for signs and symptoms of arrhythmia, an intermediate group of opportunistic screening was defined. Created in Excel, Microsoft Office 365

The above search strategy led to 626 studies in MEDLINE (via PubMed), 1227 in EMBASE and one reference in the Cochrane Database of Systematic Reviews on April 1, 2022, updated November 21, 2022. One study was manually added. After meticulous revision of studies that reported devices and AF screening methods, we finally included 24 studies for qualitative and quantitative analyses (Fig. 1). Eight controlled and 16 cohort studies were identified. Of these, 21 studies were prospectively and three retrospectively designed. All controlled trials were eligible for quantitative analysis. Due to heterogeneous performance of AF screening, the 24 studies were grouped according to screening strategy: four studies with high-quality screening, ten with opportunistic screening and ten with low-quality screening (Fig. 2). According to the criteria of the Oxford University, these references represent a level of evidence of 2 and 3 [16].

### Quantitative comparison of AF incidence in PFO closure vs. medication

Six randomised controlled (RCT) and two non-randomised controlled trials were eligible for quantitative analysis of AF incidence after percutaneous PFO closure [3, 4, 7, 10, 19–22]. These eight studies reporting on 3745 patients included exclusively patients after cryptogenic stroke and TIA with proven PFO. Patients undergoing percutaneous PFO closure were compared to conservative comparator treatment. Medication strategies included either antiplatelet therapy [10, 19, 22] or oral anticoagulants (OAC) in a minority of cases. The results were published from 2008 to 2021. The mean age of the patients within each trial was 42.9–51.8 years and 45–65% were male. Only the two oldest trials had a systematic approach in AF detection using event loop recorders, but a limited follow-up period of 6 and 12 months [7, 19]. The others had structured ECGs and rarely Holter-ECG for symptomatic patients, though, for a long mean follow-up duration of 2.0 to 5.9 years. Burow et al., PC, RESPECT and DEFENSE-PFO used the Amplatzer PFO device [4, 19–21], CLOSURE I the Starflex (NMT) [3], REDUCE the Gore Helex and Cardioform [10]. In CLOSE [22] and Bonvini et al., the operators chose different devices that were most suitable.

**Fig. 3:**
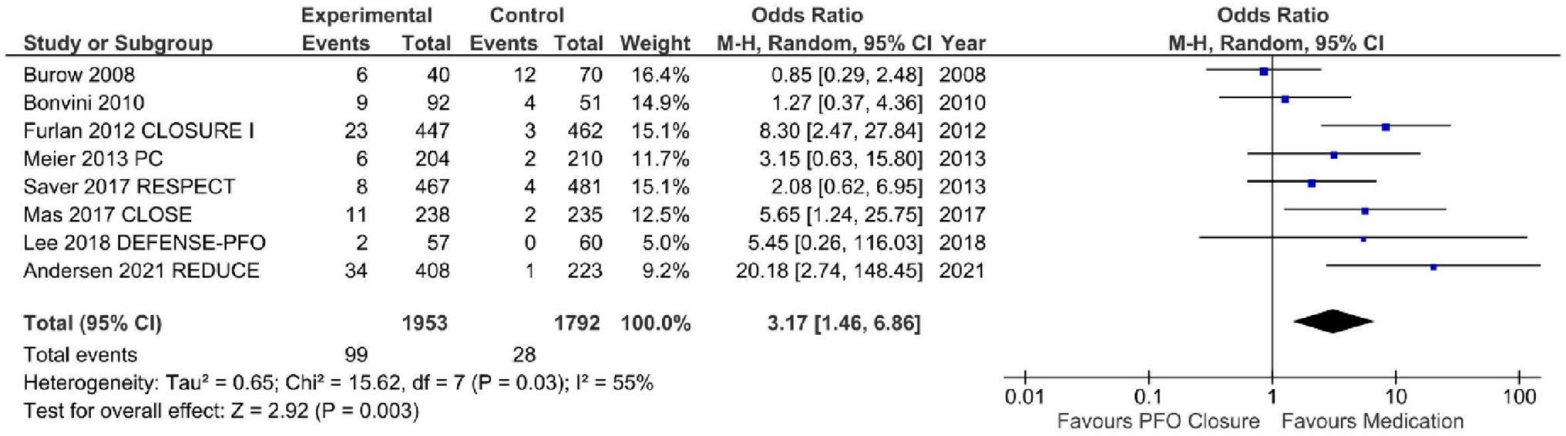
AF incidence in comparison of controlled trials A meta-analysis of all eligible controlled trials was performed. The defined primary outcome AF incidence was significantly higher in the interventional arm compared to the conservative arm (OR 3.14, CI 1.46–6.86, *P* = 0.03). Created in Review Manager 5.4

Pooled data analyses of these eight studies showed 99 AF events within the PFO closure group consisting of 1,953 patients in comparison to 28 events within in the placebo-controlled group consisting of 1,792 patients. The AF incidence was 5.1% vs. 1.6% in favour for conservative treatment (*N* = 8 trials, OR 3.17, 95% CI 1.46–6.86, *P* = 0.03, *I*^2^ = 55%, Fig. 3).

### Comparison of AF screening methods

According to the study designs, different approaches in detection of AF after percutaneous PFO closure were chosen. Amongst the 24 studies, the screening of AF was conducted in distinctive levels of quality.

#### Surveillance strategy with high-quality AF screening

Four studies reporting on 362 patients after percutaneous PFO closure were systematically screened by Holter ECG and/or ELR (Table 1) [7, 11, 19, 23]. Burow et al. used ELR for detection of AF 3 and 6 months after the procedure and Bonvini et al. did the analyses on day 1 and after 6 and 12 months. Ates et al. and Leclercq et al. performed AF Screening with Holter ECG, mainly on the date of the procedure and after 6 months, Leclercq et al. additionally after 1 month. Indication for PFO occlusion was cryptogenic stroke or TIA after standardised work-up protocol to rule out atrial fibrillation except for five patients. Two trials included four patients with decompression disease [7, 11], and one trial enrolled a diving patient with PFO closure for prophylactic treatment [7]. Overall, the procedural AF incidence until one month was 4.2–6.5%. As Burow et al. only reported results cumulative after 6 months, the AF incidence was 15% with an odds ratio of 0.85 (95% CI 0.29, 2.48). Only Bonvini et al. detected 3 AF events after 1 month resulting in an incidence of 3.3% and an odds ratio of 1.27 (95% CI [0.37, 4.36]).

**Table 1:** Surveillance strategy with systematic high-quality AF-screening

Study	Size	AF-screening	Device (*n*)	AF < 1 m	AF ≥ 1 m
Burow et al. (2008) [19]	110	ELR 3 and 6 m	Amplatzer PFO (40)	nd	6 (15%)
Bonvini et al. (2010) [7]	143	Holter/ELR 0, 6 and 12 m	Most suitable (92)	6 (6,5%)	3 (3,3%)
Ates et al. (2015) [23]	47	Holter 0, 6 m	Amplatzer PFO (34), Occlutech Figulla (13)	2 (4,2%)	0
Leclercq et al. (2021) [11]	62	Holter 0, 1, 6 m	Amplatzer PFO (42), Amplatzer Cribiform (15), Occlutech PFO (5)	3 (4,8%)	0

*AF* atrial fibrillation, *ELR* external loop recorder, *m* month, *nd* not done

#### Surveillance strategy with opportunistic AF screening

Ten studies reporting on 4853 patients after percutaneous PFO closure screened both systematically and non-systematically mostly by ECG and symptom-driven Holter-ECG (Table 2) [5, 6, 8, 10, 24–29]. Few patients had other indications than stroke or TIA, e.g. migraine, platypnea-orthodeoxia syndrome or decompression sickness (*n* = 103). The AF incidence until the first follow-up at one month presented a range of 0 to 1.8% in the smallest trials [6, 28, 29]. Larger trials with a sample size of more than 600 demonstrated an AF incidence of 2.4–6.6% in early onset. The late onset ranged from 0.4 to 4.9% [5, 8, 27]. These trials had a long mean follow-up duration of 3.2 to 12.3 years. Therefore, most AF events were detected in those studies. Wagdi et al. reported an AF incidence of 10.3%, increased by a subgroup of 20 Occlutech devices with an AF event rate of 25%.

**Table 2:** Surveillance strategy with opportunistic AF-screening

Study	Size	AF-screening	Device (*n*)	AF < 1 m	AF ≥ 1 m
Alaeddini et al. (2006) [24]	71	ECG 1, 6, 12 m; symptoms Holter	CardioSEAL (67), Amplatzer PFO (4)	2 (2.8%)	1 (1.4%)
Kiblawi et al. (2006) [25]	456	ECG 1, 6 m; symptoms Holter	CardioSEAL (456)	16 (3.5%)	0
Staubach et al. (2009) [5]	1349	ECG 1, 3, 6 m, yearly; symptoms ECG/Holter	Amplatzer (535), Helex (379), Starflex (270), Premere (131), Sideris (9), Asdos (9), CardioSEAL (7), Angelwings (5), PFO Star (4)	33 (2.4%)	20 (1.5%)
Wagdi (2010) [6]	68	ECG 1, 6 m; symptoms Holter	Amplatzer (28), Occlutech (20), Solysafe (9), Premere (8), Cardia (3)	1 (1.8%)	7 (10.3%)
Bronzetti et al. (2011) [26]	276	ECG 1, 6, 12 m, yearly; symptoms ECG/Holter	Amplatzer PFO (174), CardioSEAL (57), Premere (28), Helex (7), Cardia (5), Biostar (5)	10 (3.6%)	1 (0.4%)
Hornung et al. (2013) [27]	660	ECG 1, 6 m; symptoms ECG/Holter	Amplatzer PFO (220), CardioSEAL (220), Helex (220)	18 (2.7%)	22 (3.3%)
Geis et al. (2015) [29]	41	ECG 6 w, 6 m, Holter symptoms	Cardioform Septal (41)	0	2 (4.9%)
Rigatelli et al. (2016) [8]	1000	ECG 1, 6, 12 m, yearly; Holter 1 m	Amplatzer PFO (463), Amplatzer Cribiform (420), Premere (95), Biostar (22)	47 (4.7%)	5 (0.5%)
He et al. (2020) [28]	268	ECG 1, 3, 6, 12 m; Holter 1 m + symptoms	Amplatzer PFO (268)	1 (0.4%)	1 (0.4%)
Andersen et al. (2021) [10]	664	Holter; ECG 1, 6, 12, 24 m	Helex (158), Cardioform Septal (250)	27 (6.6%)	7 (1.7%)

*A* amplatzer, *AF* atrial fibrillation, *B* biostar, *CS* CardioSEAL, *DU* device unclear, *H* helex, *m* month, *O* occlutech, *P* premere, *Si* sideris, *So* solysafe, *St* starflex

#### Surveillance strategy with low-quality AF screening

Ten studies reporting on 4764 patients after percutaneous PFO closure followed a low-quality screening by 12-lead ECG (Table 3) [3, 4, 20–22, 30–34]. The mean follow-up period varied from 19.3 months to 5.9 years. Mostly, neurological patients after ischaemic work-up were included, few patients (*n* = 9) with migraine, decompression sickness and diving disease in two trials. The early onset period revealed a range of AF incidence from 0.5 to 4.3%, whereas the highest rates were reported in the structured RCT protocols [3, 4, 20, 22] with more than half of the total events. AF incidence in late onset was from 0.2 to 2.8%.

**Table 3:** Surveillance strategy with low-quality AF-screening

Study	Size	AF-screening	Device (*n*)	AF < 1 m	AF ≥ 1 m
Furlan et al. (2012) [3]	909	ECG 0, 1, 6, 12, 24 m	Starflex (447)	14 (3.1%)	9 (2.0%)
Heinisch et al. (2012) [31]	407	ECG 1, 6 m, yearly	Helex (404)	n n	9 (2.2%)
Kefer et al. (2012) [30]	287 (PFO 175)	ECG 1, 6, 12 m, yearly	Cardia (118), Amplatzer PFO (101), CardioSEAL (24), Occlutech (15), Helex (10)	0	1 (0.6%)
Stanczak et al. (2012) [32]	264	ECG 1, 3, 6, 12 m, yearly	Premere (263)	6 (2.3%)	2 (0.8%)
Meier et al. (2013) [4]	414	ECG 0, 6 m, annually	Amplatzer PFO (204)	1 (0.5%)	5 (2.5%)
Scacciatella et al. (2016) [33]	458	ECG 1, 6, 12 m, yearly	Amplatzer PFO, Amplatzer Cribiform	5 (1.1%)	11 (2.4%)
Mas et al. (2017) [22]	663	ECG every 6 m	Amplatzer PFO (121), Cardia (31), Premere (22), CardioSEAL (21), Amplatzer cribiform (15), Occlutech (15), Atriasept II (3), Amplatzer ASD (2), Occlutech Flex II (2), Cardioform Septal (2), Occlutech ASD (1)	10 (4.3%)	1 (0.2%)
Saver (2017) [20]	980	ECG 1, 6, 12, 18, 24 m	Amplatzer PFO (467)	7 (1.5%)	1 (0.2%)
Lee et al. (2018) [21]	120	ECG 1, 3, 6, 12, 24 m	Amplatzer PFO (120)	1 (0.8%)	1 (0.8%)
Scacciatella et al. (2018) [34]	374	ECG 1, 6, 12 m, yearly	Amplatzer PFO (359)	2 (0.5%)	10 (2.8%)

*A* amplatzer, *AF* atrial fibrillation, *m* month, *n n* nullum nomen

### Devices and AF incidence

Throughout the 24 studies, the Amplatzer PFO Device (Abbott) was mostly used in 49.5% of all cases. Helex (Gore), CardioSEAL (NMT), Starflex (NMT) and Premere (St. Jude Medical) were second, third, fourth and fifth with 15.4%, 11.2%, 9.4% and 7.2%, respectively. Further devices Cardioform Septal (3.8%, Gore), Ultrasept (2.0%, Cardia), Figulla (0.9%, Occlutech), BioSTAR (0.4%, NMT), Sideris and Solysafe (each 0.1%, the latter Swissimplant AG) were not compared due to low implantation numbers (*n* = 565, 7.4%). Additional information is available in the supplementary table 1. Due to the varying study designs, a direct head-to-head comparison was not feasible. Ranges of AF incidence and total events are also divided in early and late onset. Not all studies reported AF events for the used devices (*n* = 16).

The average AF incidence can be compared amongst the frequently used devices (Table 4). Early-onset AF was reported in 0.9% of cases for Amplatzer, 0.7% for Helex, 4.2% for CardioSEAL, 4.5% for Starflex and 2.1% for Premere. Late-onset AF incidence was reported in 1.5% of cases for Amplatzer, 1.3% for Helex, 2.0% for CardioSEAL, 2.5% for Starflex and 0.7% for Premere.

**Table 4:** Comparison of frequently used devices

Device	Ratio (%)	AF incidence < 1 m (%)	AF incidence ≥ 1 m (%)
Amplatzer PFO	49.5	0.9	1.5
Helex	15.4	0.7	1.3
CardioSEAL	11.2	4.2	2.0
Starflex	9.4	4.5	2.5
Premere	7.2	2.1	0.7

### AF management

Overall, 307 patients had documented AF episodes during follow-up (Table 5). Of these, 172 had detailed information of AF management. Spontaneous conversion was documented in 44 patients. Rhythm control strategy by either medical or electrical cardioversion was chosen in 111 patients. Two patients underwent catheter ablation, and two other patients were treated with surgical LA ablation. Rate control strategy was preferred in 17 patients. In 72 individuals were data on treatment with OAC after detection of AF available, 56 patients on long-term therapy.

**Table 5:** AF Incidence, Therapy and Anticoagulation

Study	AF < 1 m	AF ≥ 1 m	Spontaneous c	Medical c	Electrical c	Rate control	Ablation	Surgery	OAC < 6 m	OAC ≥ 6 m	TIA	Stroke
Alaeddini et al. (2006) [24]	2	1	1	0	0	1	0	1	n n	n n	0	0
Kiblawi et al. (2006) [25]	16	0	0	11	3	0	0	0	3	0	4	3
Burow et al. (2008) [19]	0	6	n n	n n	n n	n n	n n	n n	0	4	0	0
Staubach et al. (2009) [5]	33	20	7	22	4	0	0	0	0	22	3	0
Wagdi (2010) [6]	1	6	0	3	2	4	0	0	3	1	0	0
Bonvini et al. (2010) [7]	6	3	1	5	0	2	0	1	0	2	0	0
Bronzetti et al. (2011) [26]	9	1	5	1	0	0	0	0	n n	n n	n n	n n
Furlan et al. (2012) [3]	14	9	n n	n n	n n	n n	n n	n n	n n	n n	13	12
Heinisch et al. (2012) [31]	0	9	5	1	1	0	0	0	n n	n n	4	6
Kefer et al. (2012) [30]	n n	n n	n n	n n	n n	n n	n n	n n	n n	n n	0	5
Stanczak et al. (2012) [32]	6	2	2	0	1	2	1	0	0	2	3	6
Hornung et al. (2013) [27]	18	22	0	25	0	0	0	0	n n	n n	10	12
Meier et al. (2013) [4]	1	5	2	2	1	0	0	0	n n	n n	5	1
Geis et al. (2015) [29]	0	2	2	0	0	0	0	0	0	2	0	0
Scacciatella (2015)	5	11	0	0	2	0	0	0	0	11	6	2
Ates et al. (2015) [23]	2	0	2	0	0	0	0	0	n n	n n	5	2
Rigatelli et al. (2016) [8]	47	5	0	3	1	0	1	0	n n	n n	n n	n n
Mas et al. (2017) [22]	10	1	n n	n n	n n	n n	n n	n n	7	3	8	0
Saver (2017) [20]	7	1	n n	n n	n n	n n	n n	n n	n n	n n	17	18
He et al. (2020) [28]	1	1	1	1	0	0	0	0	n n	n n	2	0
Lee et al. (2018) [21]	n n	n n	n n	n n	n n	n n	n n	n n	n n	n n	0	0
Andersen et al. (2021) [10]	27	7	13	9	9	8	0	0	6	7	0	6
Leclercq et al. (2021) [11]	3	0	3	0	0	0	0	0	0	2	0	0
Total	201	112	44	83	24	17	2	2	16	56	76	73

*AF* atrial fibrillation, *c* cardioversion, *m* month, *nullum* nomen

### Incidence of stroke and TIA

For the whole study cohort of 7643 patients, the cumulative incidence of TIA and stroke was 1.9%, summarising all events since implantation. Only four studies [3, 10, 30, 32] reported six patients with ischaemic events after detected AF episodes.

## Discussion

To our knowledge, this is the first meta-analysis comprehensively assessing the effect of PFO closure on AF incidence compared to conservative treatment. The main and novel findings of pooled data analyses were:

1. Higher AF incidence following percutaneous PFO closure compared to medication
2. Controlled studies with ELR screening were neutral in AF detection
3. Higher AF incidence in systematic vs. opportunistic and low-quality screening groups
4. Starflex and CardioSEAL seem to have higher AF incidence

Meta-analysis of the randomised and non-randomised controlled trials showed higher likelihood for AF in the interventional arm following PFO closure. Besides procedural monitoring, the screening method of AF was, especially in the RCTs, of reduced quality with 12-lead ECG and symptom-led Holter-ECG. The two small, controlled trials pursued a high-quality AF screening and showed an equal proportion of AF events in both treatment arms, 15 vs. 17% [19], 10 vs. 8% [7]. The data are limited by short study durations from six to twelve months, causing an elevated heterogeneity in the present analysis. If the large RCTs were using a systematic AF screening approach, the AF rate might have been higher.

### Differences in AF surveillance strategies

Consistently, the separate tables of AF screening quality showed similar results. The high-quality studies [7, 19]had the highest AF incidences in the entire study cohort with 6.5–15%. The latter was detected in the Amplatzer device, which was in contrary associated with almost lowest AF incidence in all other trials. A recent study in patients with higher cardiovascular risk factors and age older than 55 years showed an overall high incidence of 20.9% (AF, atrial flutter and supraventricular arrhythmia) within the first month after implantation, using high-quality loop recorder screening [35]. Wagdi et al. demonstrated a presuming high rate of 25% with Occlutech device [6]. No AF was detected in two other trials [11, 30]. The device type made up 1% of all 24 studies. Despite the methodological limitations of the comparison, new onset AF is obviously more likely detected by high-quality screening method regardless of onset.

### Predictors of AF

Device association of peri-interventional AF has been previously investigated [6–8, 10, 11, 35]. Potential mechanisms are local irritation of atrial myocardium, implantation itself or device size and configuration. Higher AF risk following interventional PFO occlusion in patients with larger PFO or presence of atrial septal aneurysm was postulated by some authors [6–8, 35]. Device size, age or sex was associated with heterogeneous effects on AF incidence [8, 10, 11, 35]. To date, evaluation of predictors for AF following PFO closure is limited to few studies. Given the observed high incidence in the occlusion group, future studies should systematically investigate these risk factors for individual decision making or risk factor modification in each patient.

### Device-associated AF

Most frequently implanted devices were compared. 16 studies that documented AF events for implanted devices showed presumingly higher AF rates for CardioSeal and Starflex devices regardless of onset compared to the Premere, Helex and Amplatzer. Previous meta-analysis by Vukadinovic et al. showed lower AF incidence in Amplatzer vs. two Gore Devices in REDUCE [14]. However, Amplatzer had the highest rate in Burow et al. with 15% in ELR screening, other studies reached up to 3.6% in late onset. This rather supports a high-quality screening strategy and underlying mechanisms of AF development [36].

### Clinical implications of AF detection

**Fig. 4:**
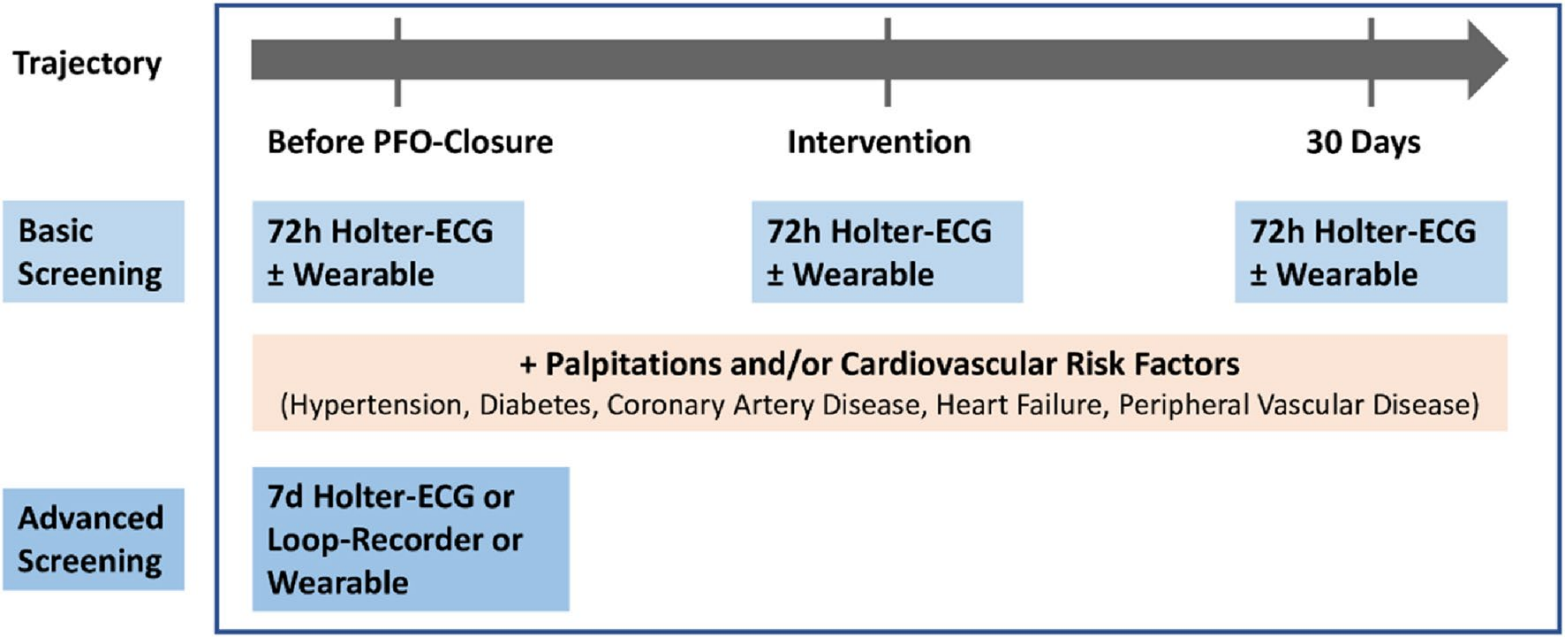
AF Rule-Out Strategy. Based on the results, a possible AF rule-out strategy was proposed, depicted as a trajectory of AF screening before, during and after the procedure. In the presence of additional cardiovascular risk factors or symptoms, an advanced AF screening should be performed before the implantation. Created in PowerPoint, Microsoft Office 365

Occult AF might play a major role, former screening strategies were less continuous. 25% of patients with cryptogenic stroke have undetected AF [37]. However, a temporal relationship between stroke and AF onset is difficult as shown by device-monitored patients suffering from strokes and recording AF. In ASSERT, subclinical AF was detected in 8% preceding their stroke, 16% after the event [38]. Therefore, thorough screening according to the guidelines before, during and after percutaneous PFO closure is recommended. Due to the results of the highest AF incidence within one month after implantation, the hypothesis of temporary inflammation seems to be more likely to explain the new onset of AF than a chronic condition. Current guidelines recommend at least 24 h of short-term ECG followed by 72 h of continuous ECG in patients with acute stroke or TIA and previously unknown AF. In this population of cryptogenic strokes, a more extensive AF rule-out must be performed, and a PFO might appear as an innocent bystander. In these 24 trials, only 15 gave details about their rule-out strategy and 12 fulfilled the guideline recommendations. In conclusion, we propose a standardised AF rule-out protocol (Fig. 4). Based on the results, we would recommend a basic screening of 72 h Holter-ECG and, if available, a wearable to detect AF before, during and after the procedure. In the presence of additional cardiovascular risk factors or symptoms, an advanced AF screening with 7 days Holter-ECG or Loop Recorder should be performed before the implantation.

Stroke and Prevention.

The incidence of recurrent neurological events was 1.9% in this study group over the whole duration. OAC can be administered as an alternative. Several studies were performed in the field of embolic stroke of undetermined source. Four studies showed neutral results in recurrent stroke reduction comparing ASS to OAC, including Warfarin [39], Rivaroxaban [40], Dabigatran [41] and Apixaban [42]. Systematic AF screening with consequent OAC in prevention of stroke was lately studied in STROKESTOP [43] and LOOP [44], where no clinical benefit in primary prevention was found. However, the patients were older, and had higher cardiovascular risk profiles compared to the PFO cohort. An algorithm for post-PFO closure AF was proposed by Elgendy et al., led by differentiation in primary and secondary AF. Long-term OAC was recommended for primary AF, short-term in secondary AF [12].

### Limitations

Both, AF rule out before PFO closure and AF follow-up strategies are performed heterogeneously due to a lack of standardised protocols. In one-third of the trials, the AF rule-out strategy was not documented in detail. Moreover, large RCTs on differentiation of AF surveillance strategies are missing. Device comparisons were statistically not possible according to different study designs.

## Conclusion

Patients after percutaneous PFO closure had a higher AF incidence compared to the conservative arm. Heterogeneity in surveillance and follow-up strategy limited the generalizability. Hence, in the future trials, a standardised follow-up is required for definite interpretation.

### Supplementary Information

Below is the link to the electronic supplementary material.

Supplementary file1 (DOCX 34 KB)

## Data Availability

All data were extracted from already published studies.

## Abbreviations

AF: Atrial fibrillation
ECG: Electrocardiogram
ELR: External loop recorder
OAC: Oral anticoagulation
PFO: Patent foramen ovale
RCT: Randomized controlled trial
TIA: Transient ischaemic attack

## References

[1] Bridges ND, Hellenbrand W, Latson L, Filiano J, Newburger JW, Lock JE (1992). Transcatheter closure of patent foramen ovale after presumed paradoxical embolism. Circulation.

[2] Pristipino C, Horst S, Fabrizio DA (2019). European position paper on the management of patients with patent foramen ovaleGeneral approach and left circulation thromboembolism. EuroIntervention.

[3] Furlan AJ, Reisman M, Massaro J (2012). Closure or medical therapy for cryptogenic stroke with patent foramen ovale. N Engl J Med.

[4] Meier B, Kalesan B, Mattle HP (2013). Percutaneous closure of patent foramen ovale in cryptogenic embolism. N Engl J Med.

[5] Staubach S, Steinberg DH, Zimmermann W (2009). New onset atrial fibrillation after patent foramen ovale closure. Catheter Cardiovasc Interv.

[6] Wagdi P (2010). Incidence and predictors of atrial fibrillation following transcatheter closure of interatrial septal communications using contemporary devices. Clin Res Cardiol.

[7] Bonvini RF, Sztajzel R, Dorsaz PA (2010). Incidence of atrial fibrillation after percutaneous closure of patent foramen ovale and small atrial septal defects in patients presenting with cryptogenic stroke. Int J Stroke.

[8] Rigatelli G, Zuin M, Pedon L (2017). Clinically apparent long-term electric disturbances in the acute and very long-term of patent foramen ovale device-based closure. Cardiovasc Revasc Med.

[9] Vitarelli A (2019). Patent foramen ovale: pivotal role of transesophageal echocardiography in the indications for closure, assessment of varying anatomies and post-procedure follow-up. Ultrasound Med Biol.

[10] Andersen A, Matzen KL, Andersen G (2021). Atrial fibrillation after closure of patent foramen ovale in the REDUCE clinical study. Catheter Cardiovasc Interv.

[11] Leclercq F, Odorico X, Marin G (2021). Atrial fibrillation screening on systematic ambulatory electrocardiogram monitoring after percutaneous patent foramen ovale closure: a prospective study. IJC Heart Vasc.

[12] Elgendy AY, Islam YE, Mohammad KM (2019). New-onset atrial fibrillation following percutaneous patent foramen ovale closure: a systematic review and meta-analysis of randomised trials. EuroIntervention.

[13] Oliva L, Huszti E, Barker M (2021). New-onset atrial fibrillation following percutaneous closure of patent foramen ovale: a systematic review and meta-analysis. J Interv Card Electrophysiol.

[14] Vukadinović D, Scheller B, Ukena C, Ewen S, Mahfoud F, Böhm M (2022). Device-related risk of atrial fibrillation after closure of patent foramen ovale: a systematic review and meta-analysis. Clin Res Cardiol.

[15] Moher D, Liberati A, Tetzlaff J, Altman DG (2009). Preferred reporting items for systematic reviews and meta-analyses: the PRISMA statement. BMJ.

[16] Howick J, Chalmers I, Glasziou P (2011). The 2011 Oxford CEBM evidence levels of evidence (Introductory Document).

[17] Higgins JPT, Altman DG, Gøtzsche PC (2011). The Cochrane Collaboration’s tool for assessing risk of bias in randomised trials. BMJ.

[18] Sterne JA, Hernán MA, Reeves BC (2016). ROBINS-I: a tool for assessing risk of bias in non-randomised studies of interventions. BMJ.

[19] Burow A, Schwerzmann M, Wallmann D (2008). Atrial fibrillation following device closure of patent foramen ovale. Cardiology.

[20] Saver JL, Caroll JD, Thaler DE (2017). Long-Term Outcomes of Patent Foramen Ovale Closure or Medical Therapy after Stroke. N Engl J Med.

[21] Lee PH, Song JK, Kim JS (2018). Cryptogenic stroke and high-risk patent foramen ovale: the DEFENSE-PFO trial. J Am Coll Cardiol.

[22] Mas JL, Derumeaux G, Guillon B (2017). Patent foramen ovale closure or anticoagulation vs. antiplatelets after stroke. N Engl J Med.

[23] Ateş AH, Sunman H, Aytemir K (2015). Prevention of recurrent cryptogenic stroke with percutaneous closure of patent foramen ovale; one year follow-up study with magnetic resonance imaging and Holter monitoring. Turk Kardiyoloji Dernegi Arsivi.

[24] Alaeddini J, Feghali G, Jenkins S, Ramee S, White C, Abi-Samra F (2006). Frequency of atrial tachyarrhythmias following transcatheter closure of patent foramen ovale. J Invasive Cardiol.

[25] Kiblawi FM, Sommer RJ, Levchuck SG (2006). Transcatheter closure of patent foramen ovale in older adults. Catheter Cardiovasc Interv.

[26] Bronzetti G, D'Angelo C, Donti A (2011). Role of atrial fibrillation after transcatheter closure of patent foramen ovale in patients with or without cryptogenic stroke. Int J Cardiol.

[27] Hornung M, Bertog SC, Franke J (2013). Long-termresults of a randomized trial comparing three different devices for percutaneous closure of a patent foramen ovale. Eur Heart J.

[28] He L, Cheng G, Du Y, Zhang Y (2020). Importance of persistent right-to-left shunt after patent foramen ovale closure in cryptogenic stroke patients. Tex Heart Inst J.

[29] Geis NA, Pleger ST, Katus HA, Hardt SE (2015). Using the GORE® septal occluder (GSO) in challenging patent foramen ovale (PFO) anatomies. J Interv Cardiol.

[30] Kefer J, Sluysmans T, Hermans C (2012). Percutaneous transcatheter closure of interatrial septal defect in adults: procedural outcome and long-term results. Catheter Cardiovasc Interv.

[31] Heinisch C, Bertog S, Wunderlich N (2012). Percutaneous closure of the patent foramen ovale using the HELEX® septal occluder: acute and long-term results in 405 patients. EuroIntervention.

[32] Stanczak LJ, Bertog SC, Wunderlich N, Franke J, Sievert H (2012). PFO closure with the Premere PFO closure device: acute results and follow-up of 263 patients. EuroIntervention.

[33] Scacciatella P, Meynet I, Presbitero P (2016). Recurrent cerebral ischemia after patent foramen ovale percutaneous closure in older patients: a two-center registry study. Catheter Cardiovasc Interv.

[34] Scacciatella P, Meynet I, Giorgi M (2018). Angiography vs transesophageal echocardiography-guided patent foramen ovale closure: a propensity score matched analysis of a two-center registry. Echocardiography.

[35] Guedeney P, Laredo M, Zeitouni M (2022). Supraventricular arrhythmia following patent foramen ovale percutaneous closure. J Am Coll Cardiol Intv.

[36] Won H, Carroll JD (2020). Comparative effectiveness research applied to medical devices: Which PFO closure device is the best?. Catheter Cardiovasc Interv.

[37] Kotadia ID, Sim I, Mukherjee R (2021). Secondary stroke prevention following embolic stroke of unknown source in the absence of documented atrial fibrillation: a clinical review. J Am Heart Assoc.

[38] Healey JS, Connolly SJ, Gold MR (2012). Subclinical atrial fibrillation and the risk of stroke. N Engl J Med.

[39] Mohr JP, Thompson JLP, Lazar RM (2001). A comparison of warfarin and aspirin for the prevention of recurrent ischemic stroke. N Engl J Med.

[40] Hart RG, Sharma M, Mundl H (2018). Rivaroxaban for stroke prevention after embolic stroke of undetermined source. N Engl J Med.

[41] Diener H-C, Sacco RL, Easton JD (2019). Dabigatran for prevention of stroke after embolic stroke of undetermined source. N Engl J Med.

[42] Geisler T, Keller T, Martus P, Ziemann U, Poli S (2022). Neue Daten zum embolischen Schlaganfall unbekannter Ursache. CardioNews.

[43] Svennberg E, Friberg L, Frykman V, Al-Khalili F, Engdahl J, Rosenqvist M (2021). Clinical outcomes in systematic screening for atrial fibrillation (STROKESTOP): a multicentre, parallel group, unmasked, randomised controlled trial. Lancet.

[44] Svendsen JH, Diederichsen SZ, Højberg S (2021). Implantable loop recorder detection of atrial fibrillation to prevent stroke (The LOOP Study): a randomised controlled trial. Lancet.

