# Supplementary material for: New-onset atrial fibrillation after percutaneous patent foramen ovale closure: a meta-analysis
Source: Clin Res Cardiol. 2023 Jul 29;112(12):1824–34. doi: 10.1007/s00392-023-02263-8 (PMC10697880; doi:10.1007/s00392-023-02263-8)
Supplement: Supplementary file 1 — Supplementary file1 (DOCX 34 KB) [file 392_2023_2263_MOESM1_ESM.docx]

**Supplemental material**

| Supplementary Table 1 Devices and AF incidence | | | |  |
| --- | --- | --- | --- | --- |
| Device | Total no. | Device reported | events < 1 m | events ≥ 1 m |
| Amplatzer PFO | 3784 (49.5%) | 3078 (81.3%) | 29 | 47 |
| BioSTAR | 27 (0.4%) | 27 (100%) | n n | 1 |
| Ultrasept PFO | 157 (2.0%) | 118 (75.2%) | 0 | 0 |
| CardioSEAL | 852 (11.2%) | 764 (89.7%) | 32 | 15 |
| Cardioform Septal | 293 (3.8%) | 41 (14.0%) | n n | 2 |
| Helex | 1178 (15.4%) | 609 (51.7%) | 4 | 8 |
| Figulla | 70 (0.9%) | 40 (57.1%) | n n | 6 |
| Premere | 547 (7.2%) | 422 (77.1%) | 9 | 3 |
| Sideris | 9 (0.1%) | 9 (100%) | 0 | 1 |
| Solysafe | 9 (0.1%) | 9 (100%) | 0 | 1 |
| Starflex | 717 (9.4%) | 717 (100%) | 32 | 18 |
| Total | 7643 (100%) | 5990 (78,4%) | 88 | 93 |

AF atrial fibrillation, n n nullum nomen, m month
